# Machine learning analysis of thermophysical and thermohydraulic properties in ethylene glycol- and glycerol-based SiO_2_ nanofluids

**DOI:** 10.1038/s41598-024-65411-8

**Published:** 2024-06-27

**Authors:** Suleiman Akilu, K. V. Sharma, Aklilu Tesfamichael Baheta, Praveen Kumar Kanti, Prabhu Paramasivam

**Affiliations:** 1 Department of Mechanical Engineering, Universiti Teknologi PETRONAS, Bandar Seri Iskandar, 32610 Tronoh, Perak Malaysia; 2 Center for Energy Studies, Department of Mechanical Engineering, JNTUH College of Engineering, Kukatpally, 500085 Hyderabad, Telangana India; 3 Department of Mechanical Engineering, Addis Ababa Science and Technology University, Akaki-Kality Sub-City, PO Box 16417, Addis Ababa, Ethiopia; 4 Indian Institute of Technology Madras, Chennai, 600 036 India; 5 Department of Research and Innovation, Saveetha School of Engineering, SIMATS, Chennai, Tamil Nadu 602105 India; 6 Department of Mechanical Engineering, Mattu University, 318 Mettu, Ethiopia

**Keywords:** SiO_2_, Glycerol, Ethylene glycol, Nanofluid, Heat transfer, Energy management, Machine learning, Mechanical engineering, Nanoparticles

## Abstract

The study investigates the heat transfer and friction factor properties of ethylene glycol and glycerol-based silicon dioxide nanofluids flowing in a circular tube under continuous heat flux circumstances. This study tackles the important requirement for effective thermal management in areas such as electronics cooling, the automobile industry, and renewable energy systems. Previous research has encountered difficulties in enhancing thermal performance while handling the increased friction factor associated with nanofluids. This study conducted experiments in the Reynolds number range of 1300 to 21,000 with particle volume concentrations of up to 1.0%. Nanofluids exhibited superior heat transfer coefficients and friction factor values than the base liquid values. The highest enhancement in heat transfer was 5.4% and 8.3% for glycerol and ethylene glycol -based silicon dioxide Nanofluid with a relative friction factor penalty of ∼30% and 75%, respectively. To model and predict the complicated, nonlinear experimental data, five machine learning approaches were used: linear regression, random forest, extreme gradient boosting, adaptive boosting, and decision tree. Among them, the decision tree-based model performed well with few errors, while the random forest and extreme gradient boosting models were also highly accurate. The findings indicate that these advanced machine learning models can accurately anticipate the thermal performance of nanofluids, providing a dependable tool for improving their use in a variety of thermal systems. This study's findings help to design more effective cooling solutions and improve the sustainability of energy systems.

## Introduction

Global stakeholders are becoming increasingly focused on energy management to underpin sustainability and reduce overreliance on fossil fuel consumption, which remains the leading cause of greenhouse gas emissions at record levels in the atmosphere^1,2^. Several techniques are being used to effectively reduce dangerous gas emissions in the transport, power generation, manufacturing, and built environment sectors, which include integrating renewable energy systems and applying energy-efficient technologies. The adoption of energy-efficient devices has the potential to reduce energy consumption and adverse environmental impacts to a great extent. The fact that optimizing heat exchanger design can minimize system energy consumption and hence save money on electricity was widely known^3–5^. While the early heat transfer research effort was mostly focused on changing the exchanger surface geometry via enhancement/intensification approaches, research attention shifted decades later when new heat transfer fluids were introduced termed ‘NFs’^6,7^. A fluid containing a dispersion of nanometer-sized particles improved the heat transfer of the host fluid owing to higher thermal conductivity (TC). The use of micron-sized particles was previously considered more practical, but issues such as poor suspension stability, component erosion, and blockage of flow passages limited their effectiveness^8^. NF, by contrast, presents much better dispersion stability resulting in an optimal pressure drop without clogging the heat exchanger tubes^9,10^.

Experimental analysis of convective heat transfer of NFs has been well scrutinized experimentally with different nanoparticles (NPs), including Ag, Cu, Al_2_O_3_, CuO, Fe_2_O_3_, Fe_3_O_4,_ SiO_2_, SiC, TiO_2_, ZrO_2_, CNT, and graphene. Desirable properties such as outstanding resistance to chemical attack, thermal stability, and low cost make SiO_2_ important for researchers' heat transfer considerations. Vajjha et al.^11^ reported the convective heat transfer and FF experiments with SiO_2_-EG/water NFs for concentrations of 2.0–4.0% volume over the Reynolds number (Re) range of 3000–16,000. Kulkarni et al.^12^ analyzed the effect of SiO_2_ diameter (20, 50, 100 nm) on turbulent heat transfer performance in EG/water base liquid. They demonstrated an increase in HTCs with NP diameter. Bontemps et al.^13^ determined convective heat transfer and pressure drop of SiO_2_/water flowing in a circular tube with imposed CHF for a maximum concentration of 19.0%. Compared to water, the results indicate a marked heat transfer increase of up to 80% in the turbulent range (Re = 10,000), while the enhancement was insignificant in the laminar regime (Re = 2400). Ferrouillat et al.^14^ estimated the convective HTC of SiO_2_ (22 nm)/water nanofluid in a tube at different fluid inlet temperatures of 20, 50, and 70 °C. Overall, for cooling and heating conditions, the researchers obtained heat transfer enhancements of 10% and 60% with NF concentrations of 2.3% and 18.9%, respectively, in the Re range of 200 to 10,000 compared to values with water. The heat transfer and pressure drop data for water-based SiO_2_ (30 nm) NF for flow in a helically corrugated tube with different lengths and pitch ratios has been presented by Darzi et al.^15^. Their experimental investigation covers the Re range between 5000 and 13,500 for 0.5% and 1.0% volume concentrations. They concluded that the HTC was lesser with SiO_2_ nanofluid at a higher concentration of 1.0% than at a lower particle concentration of 0.5% for flow in the plain and corrugated tube. The FF values are comparable to water when the length-to-pitch ratio increases. An experimental investigation to estimate the forced convection heat transfer characteristics of 5.0% volume SiO_2_ (127 nm)/water-based NFs in the Re range of 3000–100,000 was performed by Julia et al.^16^. A maximum heat transfer enhancement of 300% and pressure drop of up to 1000% compared to water values were observed under CHF boundary conditions. Experimental convective heat transfer and FF for SiO_2_/water nanofluid at 0.5–4.0% volume concentration were reported by Azmi et al.^17^. The fluid was maintained at a bulk temperature of 30 °C for the Reynolds range of 5000–27,000, resulting in heat transfer enhancement of about 25% at a 3.0% volume concentration. Heat transfer decreased after that as concentration increased. Conversely, the FF of NF reduced with increasing Re.

It is evident from the literature that several researchers reported experimental work in this domain and also few reported on correlation development for thermophysical properties. These nanofluids, which are made up of nanoparticles that are dispersed throughout a base fluid, have better thermal characteristics, which makes them potential candidates for enhanced heat transfer applications. Experimentation or simulation is used to gather data on critical factors such as nanoparticle concentration, temperature, fluid flow rate, and thermal conductivity^18,19^. However, when data is large and complex-nonlinear, it becomes difficult to model the data and find the correlation. The use of machine learning (ML) is an efficient method for predicting the convective heat transfer capacities of nanofluids^20,21^. The use of deep learning, and neural networks, in particular, provides a sophisticated method for locating intricate patterns within comprehensive datasets. The model is trained and tested on a split dataset, and statistics-based performance metrics are employed to evaluate the model's performance^22,23^. With the help of the latest methods in machine learning, like ensemble approaches the researchers can get a better understanding of the complex physics involved in heat transfer in SiO_2_ nanofluids as a result of the insights they receive on the significance of several factors^24–26^. In addition to aiding in evaluation, machine learning models may also be of use in optimizing processes by adjusting factors such as nanoparticle concentration or temperature to get the desired results^27,28^. Once it has been trained, the model can generate real-time predictions, which may provide substantial insights for experimental design and process optimization.

Researchers have concentrated on determining the convection heat transfer of SiO_2_/water NFs^13–17^. While water-based NFs have shown remarkable heat transfer capability, they still have drawbacks due to base fluid characteristics, as scientific evidence shows^11^. Glycerol, like water, has long been used as a heat transfer coolant and antifreeze agent. The viscous nature of glycerol presents challenges in thermal applications where low pumping power consumption is required, affecting overall system efficiency. Previous research has primarily focused on improving thermal performance with nanofluids; however, significant gaps exist in the optimization of ethylene glycol and glycerol-based silicon dioxide nanofluids under different flow conditions and concentrations. Specifically, little is known about the comparative heat transfer performance of these nanofluids, especially at high Reynolds numbers and varying nanoparticle concentrations. This study fills these gaps by conducting a thorough comparative analysis of the heat transfer performance of silicon dioxide nanofluids with base liquids glycerol and ethylene glycol. Experiments were carried out at 80 °C with nanofluid concentrations ranging from 0 to 1% volume. The base liquids' thermophysical properties and heat transfer coefficients were analyzed and validated with standardized instruments. A series of heat transfer experiments with nanofluids were then carried out for Re numbers as high as 13,000 under constant heat flux boundary conditions.

To improve predictive accuracy and create a transparent prediction model for this complex engineering problem, five different ML approaches were evaluated for prognostic efficiency. The methods used were LR, RF, XGBoost, AdaBoost, and DTs. To determine the most effective ML techniques, a thorough evaluation was performed using statistical techniques and scientific visualization tools such as violin plots and Taylor's diagrams. This study is unique in that it focuses on comparing the thermophysical and heat transfer properties of glycerol and ethylene glycol-based silicon dioxide nanofluids while also using advanced machine learning models to predict their performance. This study aims to fill a research gap by providing a more in-depth understanding of the behavior of these nanofluids under various conditions, as well as developing reliable predictive models to aid in thermal system design and optimization.

## Experimental apparatus and method

### NF preparation

**Figure 1:**
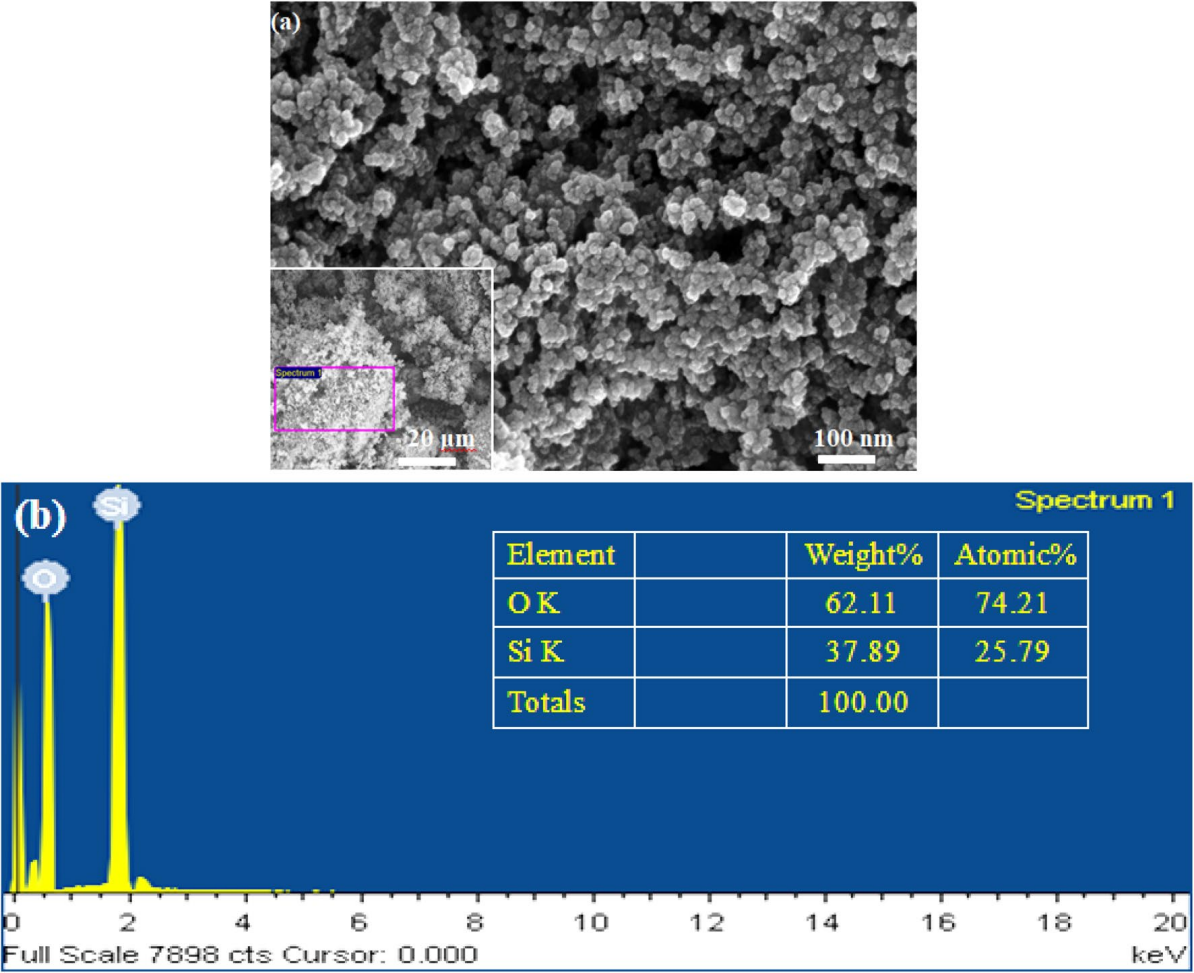
(**a**) SEM image of SiO_2_ nanoparticle and (**b**) EDX analysis of the selected area in the SEM image.

SiO_2_ NPs powder was purchased from US Research Nanomaterials Inc., USA. EG and G with 99% high purity were obtained from R&M Chemicals. The scanning electron microscopy (SEM) image of the SiO_2_ was taken using a Field Emission Scanning Electron Microscope (Supra 55VP FE-SEM, Carl Zeiss). Figure 1a depicts the SEM images. As seen, the NPs are nearly spherical shaped. The particle sizes vary between 8 and 25 nm, with an average particle size estimated as 21 nm^29^. The EDX analysis of the selected area in the SEM image is displayed in Fig. 1b. The result indicates that the composition of SiO_2_ is $100\%$ consistent with vendor specifications.

NFs are prepared in the respective base liquids, G and EG, following a two-step method^14^. Each quantity of NPs required for 0.5%, 0.75%, 1.0%, and 2.0% volume fractions was taken on an electronic balance (TLE 104E, Mettler-Toledo). The formulation was achieved by dispersing SiO_2_ in the base liquids with a magnetic agitator to stir the sample in a beaker for 30 min while adjusting the pH value. Following a similar protocol, another set of SiO_2_ NFs in a 60:40 by volume G and EG was prepared for comparison.

It is reported that NPs would agglomerate to form clusters and settle over time due to high surface energies^30^. Kinetic energy is needed to break down the particle clusters into minute sizes, according to Darzi et al.^15^. Given that, the samples have been subjected to an ultrasonic homogenization (Labsonic M, Sartorius AG) operating at a 30 kHz frequency for 2 h to improve the stability of the dispersions. The pH control is crucial in colloidal stability as it determines the suspension's isoelectric point (IEP). Highly acidic NF (low pH) can lead to corrosion with the long-term flow in pipes. The pH value of the NF samples varied between 6 and 7. The pH value was set to 10 by adding NH_3_OH to have zeta potential values away from IEP^31^. No dispersant was added during the preparation process to avoid altering the properties of the NF.

The stability of NFs was checked using Zetasizer (Nano ZSP, Malvern), which operates on the principle of dynamic light scattering to measure charge repulsion/attraction between dispersed particles. At pH 10, the dispersions had an average absolute zeta potential of − 33 and − 42 mV indicating that the SiO_2_ NFs are stable. Further, a small portion of the NFs is kept under static conditions for months and examined. G-based NFs were stable for over three months, whereas EG-based NFs were stable for up to 1 month without settling in clear storage containers.

### Thermophysical property measurement

The thermal property analyzer (KD2 Pro Decagon Devices) based on the transient hot-wire method was used to evaluate the effective TC of NFs within a specified precision of 5.0%. The KS-1 probe with a 60 mm length and 1.28 mm diameter was selected, which provides a transient line heat source. The sample temperature was controlled using an isothermal bath (Vivo-RT2, Julabo), with temperature stabilization better than ± 0.1 K. A rotational rheometer (MCR 302, Anton Paar) was used for effective viscosity measurement. A double-gap concentric cylinder was employed as the measuring geometry. A gap distance of 1.0 mm was allowed between the co-axial cylinders of the system. A Peltier thermostat controlled the cell temperature with a precision of ± 0.1 K. Repeated tests were conducted LVDV-III Ultra Programmable Rheometer. A digital densitometer (DA-645, KEM) which functions on an oscillating U-tube principle, was instrumental in determining the effective density with ± 0.00005 g/cm^3^ accuracy. Peltier thermoelectric elements enable temperature control within the measuring cell, assuring a precision below 0.03 °C. A differential scanning calorimeter (DSC Q2000, TA Instruments) analyzed the specific heat with an accuracy of 2%. Precise heat measurement was made using the standard test method (ASTM-E1269) under a high-purity nitrogen atmosphere at a 20 °C/min heating rate in the DSC furnace. The device temperature is ± 0.01 °C. A refrigeration cooling system RCS90 was used to conduct specific heat testing at different temperatures.

All devices have been calibrated with either G/EG before the measurements with NFs. The data are collected in the range of temperatures 20–80 °C at atmospheric pressure. Three readings were obtained for each sample at each temperature, and the mean value was stated.

Table 1 shows the measured properties of G and EG. Comparisons have been made with the values reported in the literature^32–35^. The Hewitt^33^ data correlates well with thermal conductivity values within a maximum of 0.25 and 0.02% deviations for glycerol and ethylene glycol, respectively. Hewitt and Cabaleiro’s specific heat data showed a 0.9 and 5.6% variation, while the Lide thermal conductivity data deviated from the measured values by 1.3 and 0.6%. A maximum deviation in viscosity of 5.9 and 1.1% for G and EG was observed when compared with Lide^35^ and Quijada-Maldonado^32^, respectively, in the temperature range of 25 to 80 °C. The overall deviation of the calibration results in all experiments was better than 6% from the reference values. Table 2 presents the uncertainty of the measured thermophysical properties of glycerol (G) and ethylene glycol (EG) in percentage.

**Table 1:** Thermophysical properties of measured base liquids.

Base liquid	Temperature (^o^C)	Thermal conductivity (W/mK)	Viscosity (mPa s)	Density (kg/m^3^)	Specific heat (J/kg K)
Glycerol	25	0.2812	912	1260	2400
	30	0.2814	598	1256	2411
	40	0.2824	283	1249	2467
	50	0.2835	143	1241	2528
	60	0.2846	81.1	1235	2580
	70	0.2859	49.4	1227	2627
	80	0.2873	31.2	1221	2673
Ethylene glycerol	25	0.2511	16.3	1110	2210
	30	0.2520	13.4	1107	2260
	40	0.2546	9.2	1100	2300
	50	0.2563	6.6	1092	2390
	60	0.2582	4.7	1085	2450
	70	0.2602	3.5	1079	2520
	80	0.2621	2.9	1072	2580
Glycerol/ethylene glycerol 60:40 vol.%	25	0.2620	123	1196	2280
	30	0.2637	95.7	1193	2310
	40	0.2661	56.1	1187	2380
	50	0.2686	32.7	1180	2430
	60	0.2725	20.2	1175	2490
	70	0.2761	14.6	1169	2560
	80	0.2791	10.4	1163	2610

**Table 2:** Instruments and uncertainty of experimental parameters.

Property/parameter	Instrument	Uncertainty approach	% Uncertainty
Thermal conductivity	Decagon KD2 Pro	Standard uncertainty	2.4
Viscosity	MCR 302	Standard uncertainty	4.1
Density	DA645	Standard uncertainty	0.1
Specific heat	TA instruments Q2000	Standard uncertainty	1.8
Temperature (bulk)	Thermocouple, K-type	Least count	0.16
Temperature (wall)	Thermocouple, K-type	Least count	0.18
Voltage	Voltmeter	Least count	0.004
Current	Ammeter	Least count	0.34
Flow rate	Imf vortex flow meter SV4200	Least count	1.64
Pressure drop	Keyence pressure sensor GP-100	Least count	4.12
Reynolds number	Derived	Error propagation	4.41
Heat flux	Derived	Error propagation	0.34
Nusselt	Derived	Error propagation	2.42
Heat transfer coefficients	Derived	Error propagation	0.34
Friction factor	Derived	Error propagation	4.43

**Figure 2:**
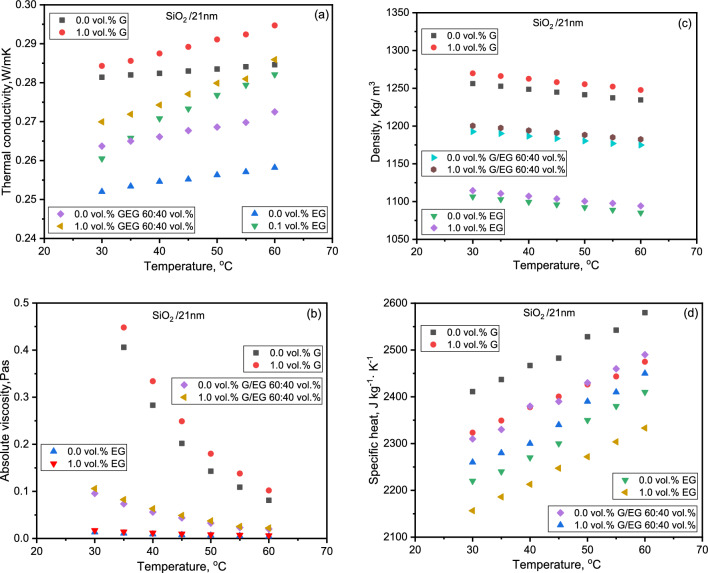
Variation of (**a**) thermal conductivity, (**b**) viscosity, (**c**) density, and (**d**) specific heat with temperature for the three nanofluids.

A comparison of the thermophysical properties of SiO_2_ NFs with the base liquid is shown in Fig. 2a–d. It was established that the TC enhancement was highest at 1.0% concentration with values of 4.2% and 10.7%, respectively, for G and EG-based NFs. The NF exhibited Newtonian behavior with viscosity independent of the shear rate in a similar manner to that evidenced by Tadjorodi et al.^36^ and Żyła and Jacek^37^. The viscosity of SiO_2_ NFs increased by 27% and 33% compared to base liquids G and EG. The suspension density increased by nearly 2% approximately. SiO_2_ NFs exhibited lower values of effective specific heat than their base fluids. Meanwhile, the specific heat decreased by nearly 2.7% and 1.5%, over a temperature range of 25 to 80 °C. Further, the measured density values of NFs are consistent with the calculated values using the mixing theory relation within 1.0% deviation in each case. The specific heat data deviated by 3% from the classical thermal equilibrium model. Escher et al.^38^ have reported a good agreement for density and specific heat values with mixture relations for water-based SiO_2_ NFs for a concentration of up to 31%.

The experimental data for the measured SiO_2_ NFs thermal conductivity, viscosity, density and specific heat was regressed to generate an empirical Eqs. (1)–(16) as a function of particle volume concertation (φ) and temperature (T) for further analysis of heat transfer as follows:

#### SiO_2_-EG nanofluid

1 $${\rho }_{{SiO}_{2}-EG}=0.99932{\left(1+\frac{\varphi }{100}\right)}^{0.83271}{\left(1+\frac{T}{80}\right)}^{0.0017}\cdot {\rho }_{EG}$$

2 $${\rho }_{EG}=1127.2-0.70070\cdot T$$

3 $${c}_{p, { SiO}_{2}-EG}=0.96805{\left(1+\frac{\varphi }{100}\right)}^{0.01}{\left(1+\frac{T}{80}\right)}^{0}\cdot {c}_{p, EG}$$

4 $${c}_{p, EG}=2039.5+6.8022\cdot T$$

5 $${k}_{{SiO}_{2}-EG}=0.9715{\left(1+\frac{\varphi }{100}\right)}^{2.3452}{\left(1+\frac{T}{80}\right)}^{0.101}\cdot {k}_{EG}$$

6 $${k}_{EG}=0.2462-0.0002\cdot T$$

7 $${\mu }_{{SiO}_{2}-EG}=0.9378{\left(1+\frac{\varphi }{100}\right)}^{10.943}{\left(1+\frac{T}{80}\right)}^{0.1988}\cdot {\mu }_{EG}$$

8 $${\mu }_{EG}=0.0323\cdot exp\left(-0.031\cdot T\right)$$

#### SiO_2_-G nanofluid

9 $${\rho }_{{SiO}_{2}-G}=1.0019{\left(1+\frac{\varphi }{100}\right)}^{0.96571}{\left(1+\frac{T}{80}\right)}^{0}\cdot {\rho }_{G}$$

10 $${\rho }_{G}=1277.2-0.71077\cdot T$$

11 $${c}_{p, { SiO}_{2}-G}=0.96805{\left(1+\frac{\varphi }{100}\right)}^{0.01}{\left(1+\frac{T}{80}\right)}^{0}\cdot {c}_{p, G}$$

12 $${c}_{p, G}=2258.8+5.191\cdot T$$

13 $${k}_{{SiO}_{2}-G}=0.97106{\left(1+\frac{\varphi }{100}\right)}^{2.3450}{\left(1+\frac{T}{80}\right)}^{0.068}\cdot {k}_{G}$$

14 $${k}_{G}=0.2779-0.0001\cdot T$$

15 $${\mu }_{{SiO}_{2}-G}=0.93782{\left(1+\frac{\varphi }{100}\right)}^{10.943}{\left(1+\frac{T}{80}\right)}^{0.1988}\cdot {\mu }_{G}$$

16 $${\mu }_{G}=3.7854\cdot exp\left(-0.062\cdot T\right)$$

With the parameter units $\varphi$ (%) and $T$ (^o^C).

### Heat transfer test setup

**Figure 3:**
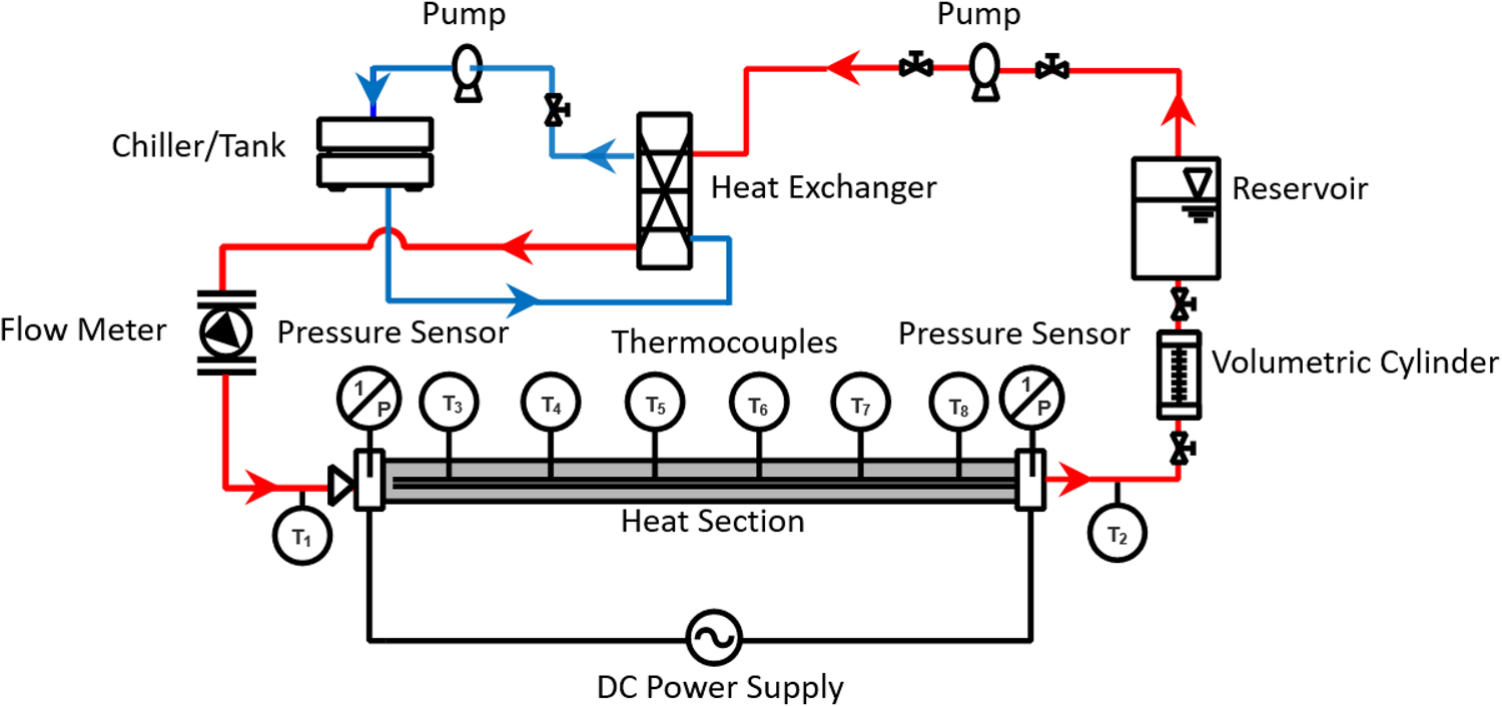
Schematic layout of experimental test-up.

The experimental system has a closed-loop design composed of five essential components: the test tube, power supply, cooling arrangement, measurement system, and data acquisition. The experimental setup schematic diagram is depicted in Fig. 3. The test section involves a single stainless-steel tube with a bell-mouth entry, thermocouples, and split heaters. The test section consists of a tube 1.0 m long with outer and inner diameters of 6.35 and 4.57 mm, respectively. The outer tube surface is embedded with two-cylinder split-body electric heaters. Each heater, with a 750 W maximum power rating, is wrapped with ceramic fiber insulation and connected to a variable transformer. Eight K-type thermocouples have been used to quantify and record the temperatures at different places. Two of the thermocouples are spot welded to the test section, each at an axial distance of 150 mm from either end of the tube, to measure the wall temperature; four thermocouples at equidistant positions of 100 mm from the tube end are located to measure the surface temperatures and two thermocouples at the inlet and outlet of the test section. All thermocouples were calibrated within a normal of ± 0.1 °C.

The cooling unit comprises a chiller, circulating pump, water tank, and temperature control system. The chiller, rated at 0.74 kW, was connected to the plate-type heat exchanger to regulate the fluid temperature at the test section inlet. A 3.0 hp horizontal multistage pump (AB, Teral) circulated the fluid in the test section. A digital vortex flow meter (SV4200, IMF) with a range of 1–20 LPM is utilized to quantify the fluid flow. A volumetric cylinder made of acrylic of 1.0 L capacity with scale graduation was connected at the test-section exit as a working fluid reservoir to check the flow rate of the fluid visually. Two absolute pressure transducers (GP-100, Keyence) were installed at the pipe inlet and outlet to measure the pressure drop (∆P) across the test section. The calibration range of the sensors from 0 to 10 MPa is ± 1%. All measuring instruments in the circuit were connected to the data logger for recording output signals.

The experiments were undertaken between 6 and 12 LPM flow rates corresponding to flow Reynold numbers of 1200–22,000, while the working fluid temperature is maintained at 80 °C. All the readings are recorded at steady-state conditions. The circuit is cleaned with water and air-dried between successive experiments.

### Data analysis

The heat energy ${Q}_{i}$ provided to the working fluid in the heat section is a function of electric current $I$ and voltage:

17 $${Q}_{i}=IV$$

Simultaneously, the rate of heat transfer ${Q}_{a}$ was evaluated from the mass flow rate $\dot{m}$ and the fluid temperatures at the inlet and outlet of the tube:

18 $${Q}_{a}=\dot{m}{c}_{p}\left({T}_{o}-{T}_{i}\right)$$

Under a steady state, the energy available with the hot fluid exiting the test section should equal the heat removed by the cooling liquid in the chiller. Newton's law of cooling relation is used to evaluate the convective HTC as follows:

19 $${h}_{exp}=\frac{{Q}_{a}}{A\left({T}_{w}-{T}_{b}\right)}$$

where the *A*, ${T}_{w}$ and ${T}_{b}$ are surface area, wall temperature and the fluid bulk or average temperature, respectively computed as:

$$A=\pi DL, \;\;\; {T}_{w}=\frac{{T}_{1}+{T}_{2}}{2}, \;\;\; {T}_{b}=\frac{{T}_{i}+{T}_{o}}{2}$$

The average Nusselt number was estimated based on the convective HTC $h$, tube diameter $D$, and TC of the fluid $k$ as

20 $${Nu}_{exp}=\frac{{h}D}{k}$$

Furthermore, the turbulent HTC can be considered from Dittus–Boelter equation in the following form:

21 $$Nu=c{\left(Re\right)}^{m}{\left(Pr\right)}^{n}$$

where *c*, *m,* and *n* represent the coefficients suitable for NF experimental data. The $Re$, and Prandtl number $(Pr)$ terms are defined as follows:

22 $$Re=\frac{{\rho }_{nf}uD}{\mu }$$

23 $$Pr=\frac{{\mu }_{nf}{c}_{p}}{{k}_{nf}}$$

The NF properties used for heat transfer analysis are determined at the bulk temperature. Further details on the derived equations for the thermophysical properties of the NF are given in Ref.^29^. The nondimensional FF $f$ was calculated from Darcy–Weisbach equation, which relates the ∆P, pipe length $L$, hydraulic diameter $D$, fluid density $\rho$, and average velocity $u$ as follows:

24 $$f=\frac{\Delta P}{\left(\frac{L}{D}\right)\left({\rho }_{nf}\frac{{u}^{2}}{2}\right)}$$

### Uncertainty analysis

Analysis of the experimental uncertainty was undertaken to validate the precision of the measurements. The uncertainties in heat transfer characteristics were estimated based on the error approach presented by Beckwith et al.^39^, following the protocol described in^17,40^. The instrument and uncertainties estimated from the measured parameters are presented in Table 2.

### Theoretical correlations for Heat transfer

#### Model 1

A correlation for Nusselt number of single-phase liquids under fully developed transition and turbulent flows is given by Gnielinski^41^ as:

25 $$Nu=\frac{\left({f}_{F}/2\right)\left(Re-1000\right)Pr}{1+12.7{\left({f}_{F}/2\right)}^{1/2}\left({Pr}^{2/3}\right)} [{1+(D/L)}^{2/3}]{ \left({\mu }_{w}/{\mu }_{f}\right)}^{0.14}$$

where the fanning friction factor is given as ${f}_{F}={\left(1.58\text{ln}Re-3.28\right)}^{-2}$. Equation (25) is valid in $the 2300\ge Re\ge {10}^{6}$ range.

#### Model 2

Other correlations are considered for a developing flow in a circular tube with a small velocity boundary layer thickness. Del Giudice^42^ developed a model for the developing flow heat transfer in a pipe exposed to uniform wall heat flux with the consideration of temperature dependence of viscosity and thermal conductivity as:

26a $$Nu=4.3636+\frac{0.065 {\left({X}^{*}\right)}^{-1.3}}{1+0.10 {\left({X}^{*}\right)}^{-n}}$$

where, $${X}^{*}=\frac{L}{{D}_{h}RePr}$$ $$n=0.761{\left(RePr\right)}^{0.0224}-0.000109 RePr$$

Where Pn_µ_ is viscosity Pearson number = (β ${q}_{w}^{"}$ D/k_e_); β =  − (dµ/dt)/µ; ${q}_{w}^{"}$ is the heat flux at the tube surface W/m^2^; D is the tube inner diameter; k_e_ is the TC at tube entry temperature. Equation (26a) is valid for $5.0 \le \text{ Pr }\le 100;$ 10^–4^ ≤ X^*^ ≤ X^*^_max_. The value of ${X}_{max}^{*}\frac{1}{ 4{Pn}_{\mu }}ln\left[\frac{{\left({Re}_{b}\right)}_{max}}{{Re}_{e}}\right]$ is estimated for the experimental conditions to be 0.08.

#### Model 3

Muzychka and Yovanovich^43^ presented a model for predicting Nu in the combined entrance region of a tube valid for uniform wall flux boundary conditions given by

26b $$Nu={\left[{\left(\frac{{C}_{4}f\left({p}_{r}\right)}{\sqrt{{z}^{*}}}\right)}^{m}+{\left({\left\{{C}_{2}{C}_{3}{\left(\frac{f\left({R}_{e}\right)}{{Z}^{*}}\right)}^{1/3}\right\}}^{5}+{\left\{{C}_{1}\left(\frac{f\left({R}_{e}\right)}{8\sqrt{\pi {\epsilon }^{\gamma }}}\right)\right\}}^{5}\right)}^{m/5}\right]}^{1/m}$$

where, $f\left({R}_{e}\right)=\frac{12}{ \sqrt{\epsilon \left(1+\epsilon \right)} \left[1-\frac{192\epsilon }{{\pi }^{5}}tanh\left(\frac{\pi }{2\epsilon }\right)\right]}$; $f\left({P}_{r}\right)=\frac{0.866}{ \sqrt{\epsilon \left(1+\epsilon \right)} \left[1-\frac{192\epsilon }{{\pi }^{5}}tanh\left(\frac{\pi }{2\epsilon }\right)\right]}$; $m=2.27+1.65{P}_{r}^{1/3}$; ${z}^{*}={L/{D}_{h}}{R}_{e}{P}_{r}$. For a circular tube, $\epsilon =1, {C}_{1}=3.86, {C}_{2}=1.5, {C}_{3}=0.501,$ and ${C}_{4}=2$

Equation (26b) is valid for 0 < Z^*^ < ∞ and 0.01 < Pr < ∞.

### Machine learning

The experimental data collected in the last section was employed to develop a comprehensive set of models to prognosticate the thermohydraulic behavior of Ethylene Glycol and Glycerol based non-porous SiO_2_ nanofluids. A battery of Python-based open-source libraries was used in the Jupytor environment. A total of five ML techniques were employed for the development of prediction-models in this case. The LR, RF, DT, XGBoost, and AdaBoost models were chosen for their various strengths: LR for simplicity and interpretability, RF, and XGBoost for excellent prediction accuracy, DT for simple decision-making, and AdaBoost for improving weak learners.

The LR was used to prepare the baseline model while the other four namely Random Forest (RF), Extreme gradient boosting (XGBoost), Adaptive boosting (AdaBoost), and Decision tree (DT) were compared against it. A brief description for each is provided as follows:

#### Linear regression

Linear regression (LR) is considered most basic form of supervised ML algorithm. In this case, a linear equation is fitted to the actual data for modeling the correlation with independent variables (features) and a dependent variable (target). The objective in this case is to represent their mutual relationship. It can be expressed as follows:

27 $$y= {\beta }_{0}+ {\beta }_{1}{x}_{1}+ {\beta }_{2}{x}_{2}+\dots + {\beta }_{n}{x}_{n}+ \varepsilon $$

Herein, y is the target (dependent variable), ${x}_{1}, {x}_{2}, {x}_{3}\dots .$ are features (independent variable), ${\beta }_{0}, {\beta }_{1}, {\beta }_{2}, \dots .$ are coefficients and $\varepsilon$ denotes the error.

LR algorithm is employed to locate the line which may provide the best fit to that minimize the sum of squared differences between the actual and predicted values.

#### Random Forest

Random Forest (RF) is a type of ensemble learning system. It is developed for regression type complex problems. In the training phase, RF generates a large number of decision trees, each of these is trained on a random portion of the training data and characteristics.

Let we denote, a training data set denoted as ‘X’ having n samples and m features. In this case y is the target variable, T denotes total count of decision trees in the test forest, X_i_ denotes a random subset taken out from training data X, sampled with replacement in case when i ranges from 1 to T. Similarly in case of features F_j_ denotes the random subset wherein, the j ranges from 1 to T.

In case of each decision tree i, a sample for training denotes as (X_i_, y_i_) is randomly selected from (X,y). Then a decision tree Di is trained on (X_i_, y_i_) employing a split criterion on the basis of least MSE.

The final prediction using a RF-based model is expressed as:

28 $$\widehat{y}= \frac{1}{T} \sum_{i=1}^{T}{D}_{i}(x)$$

In this case $\widehat{y}$ is the forecasted output D_i_(x) denotes the forecast from i-th decision tree for input x.

To summarize, RF regression employs the aggregate of several DTs trained on random subsets of data to generate robust and precise forecasts for regression problems.

#### Decision Tree

Decision Tree (DT) is a fundamental and flexible ML method which can be used for regression of data. DT creates a hierarchical tree framework having core nodes denoting feature-based decisions while leaf nodes represent the predicted values.

Let us denote a training data set as 'X' with n samples and m features. In this scenario, y is the goal variable, D is the DT model.

In the training phase, the DT splits the feature space recursively into subgroups on the basis of feature values. At each node, the DT selects the feature and split threshold to keep the MSE as low as possible. In the regression work, the prediction is done by traversing the tree from root to leaf node and assigning the mean value of the target variable inside the leaf node to the input sample.

Mathematically, the DT-based forecast can be expressed in simple terms as:

29 $$\widehat{y}=D(x)$$

It can be summarized that DT-based regression separates the feature space recursively and then makes forecast using the mean target variable value inside each zone, resulting in interpretable and simple regression models.

#### Extreme gradient boosting

Extreme gradient boosting (XGBoost) belongs to the gradient boosting family of ML. XGBoost is known for its exception prediction performance. In the gradient boosting process, it sequentially combines weak learners so as to form a robust prognostic model. XGBoost make use gradient descent approach for minimizing the loss function ‘L’ such that

30 $${min}_{\theta }\sum_{i=1}^{n}L\left({y}_{i},\widehat{{y}_{i}}\right)+ \sum_{k=1}^{k}\Omega ({\text{f}}_{k}) $$

In this case, the $\theta$ represents the parameters of model, whereas ${y}_{i} and \widehat{{y}_{i}}$ denotes the actual and predicted values. K denotes the number of weak learners in form of trees, and $\Omega ({\text{f}}_{k})$ denotes the regularization term applied to each tree.

XGBoost uses the additive approach for model building as:

31 $${y}_{i}= \sum_{k=1}^{k}{\text{f}}_{k}({\text{x}}_{i})$$

Herein, ${\text{f}}_{k}({\text{x}}_{i})$ represents the prediction of the k-th tree in case of i-th sample.

Regularization techniques like L1 and L2 are used for controlling the complexity of the individual trees.

32 $$\Omega \left({\text{f}}_{k}\right)= \gamma T+ \frac{1}{2} \lambda {\Vert \omega \Vert }_{2}^{2}$$

Herein, T is number of leaves in tree, $\omega$ denotes the leaf weights, and $\gamma$ and $\lambda$ are regularization parameters.

XGBoost also provides insights into feature importance by calculating the gain, which assesses the importance of each feature to the model's performance.

$$Gain= \frac{Gain \; in \;splitting \;creteria }{Number \;of \;times \; feature \; is \;used \;for \;splitting \;the \; data}$$

To summarize, XGBoost improves the model's accuracy by successively minimizing a loss function with gradient descent and incorporating regularization approaches to control model complexity. Its ability to offer feature importance and handle missing values render it an effective tool for regression problems across multiple domains.

#### Adaptive boosting

Adaptive boosting (AdaBoost) combines multiple weak leaners h(x) to form a strong predictor f(x). In mathematical terms the final predictor F(x) is a weighted sum of learners of weaker learners:

$$F\left(x\right)=\sum_{t=1}^{T}{\alpha }_{1}{h}_{1}(x)$$

Herein, T is the total number of weal learners, ${\alpha }_{1}$ is the weight allotted to the t-th weak learner, and ${h}_{1}(x)$ is the prediction of t-th weak learner.

In the training phase, AdaBoost allots weight to each instance of training $({x}_{i}, {y}_{i})$, in case when ${x}_{i}$ denotes the input while ${y}_{i}$ denotes true label. In starting phase all weights are set equally as:

${{w}_{i}}^{(1)}= \frac{1}{N}$;where N is the total count of instances.

AdaBoost fits a weak learner to the training data ${w}_{i}$ in each iteration t, it subsequently computes the weighted error ${\varepsilon }_{t}$ of each weak learner.

$${\varepsilon }_{t}= \sum_{i=1}^{N}{{w}_{i}}^{(t)}\cdot 1({h}_{t}({x}_{i}) \ne {y}_{i})$$

Herein, 1($\cdot$) is the indicator function. The weight ${\alpha }_{t}$ of the t-th weak learner can be estimated as:

$${\alpha }_{t}= \frac{1}{2}\text{ln}\left(\frac{1-{\varepsilon }_{t}}{{\varepsilon }_{t}}\right)$$

Subsequently, the AdaBoost updates of training instances on the basis of misclassification error:

$${{w}_{i}}^{(t+1)}= {{w}_{i}}^{\left(t\right)} \cdot \text{exp}(-{\alpha }_{t} \cdot {y}_{t} \cdot {h}_{i}\left({x}_{i}\right))$$

In the end, the weight of weak learners is merged to develop the final predictor, F(x). This process continues until a predetermined number of iterations are completed or the errors has been appropriately reduced^44^.

## Result and discussions

### Experimental test validations

**Figure 4:**
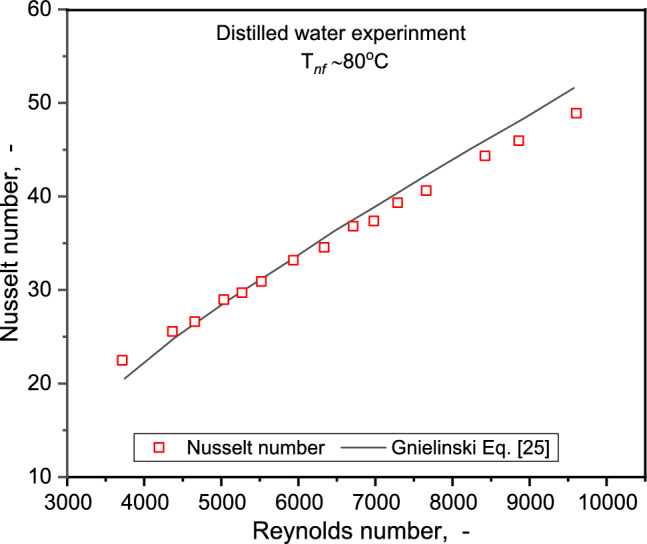
Comparison of Nu between present work and model predictions for flow of distilled water in a tube.

The experimental setup was validated by a comparison of data undertaken with water. The experimental Nusselt number values for the flow of water, as presented in Fig. 4, were compared with the correlation of Gnielinski^41^. The data correlated well with the predicted values within ± 7.4%. Further validation was carried out by contrast of the experimental FF with Eq. (27) for turbulent flow in rough pipes^45^. An excellent concordance of the experimental data was observed. Following the validation for water, heat transfer experiments progressed with the 30GW base liquid and NF concentrations of 0.25, 0.5, 0.75, and 1.0% for flow range 6 to 12 LPM.

### HTC and FF

**Figure 5:**
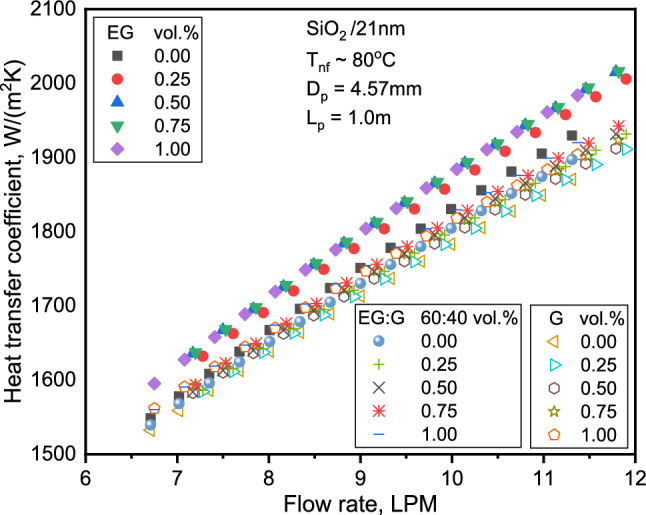
Variation of HTC with flow rate for the three NFs.

The variation of HTC with flow rate for different concentrations of SiO_2_ NFs is presented in Fig. 5. The base and NF HTC enhanced with flow rates. The increase in heat transfer reached a maximum for the three NFs at a 1.0% volume. Lower viscous SiO_2_-EG NFs exhibited significant HTC enhancement over the base liquid, compared to higher viscous SiO_2_-G NFs for similar conditions. The increase in HTC of SiO_2_-EG and SiO_2_-G NFs was determined as 5.9% and 1.9%, respectively, for a 1.0% volume fraction at a 12 LPM flow rate. This behavior could be explained by the flattening of the velocity profile and delay in boundary layer development in the fully developed region, among others^46,47^. The NF heat transfer augmentation can also be attributed to the effective TC increases caused by reduced viscosity near the wall, amplifying NPs' surface area, and particle reconfiguration^48,49^.

**Figure 6:**
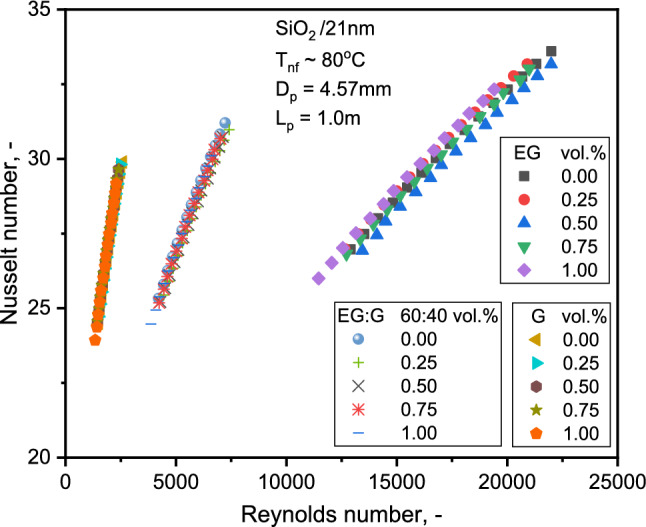
Comparison of Nu for the three NFs with Re.

Figure 6 displays the variation of Nu with Re for various SiO_2_ volume fractions. The figure shows a similar value increase over the base liquid for all nanofluids. The evolution rate is more significant for SiO_2_-EG in the turbulent flow than SiO_2_-G NFs at higher Re in the laminar flow range. Increasing the concentration enhances the Nu, possibly due to particle migration, TC enhancement, and lessening of boundary layer thickness. The enhancement in Nu at Re of 19,000 and 2,300 with 1.0% volume of the NFs are 1.4 and 1.1%, respectively. The findings are consistent with the results of SiO_2_/water in the laminar range, where the heat transfer improvement is relatively minuscule with growth in Re^38,50^.

**Figure 7:**
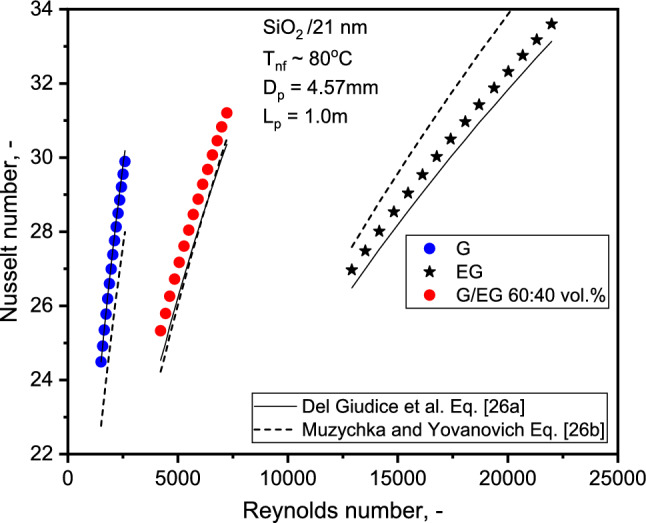
Comparison of Nu for the three base liquids with theory.

**Figure 8:**
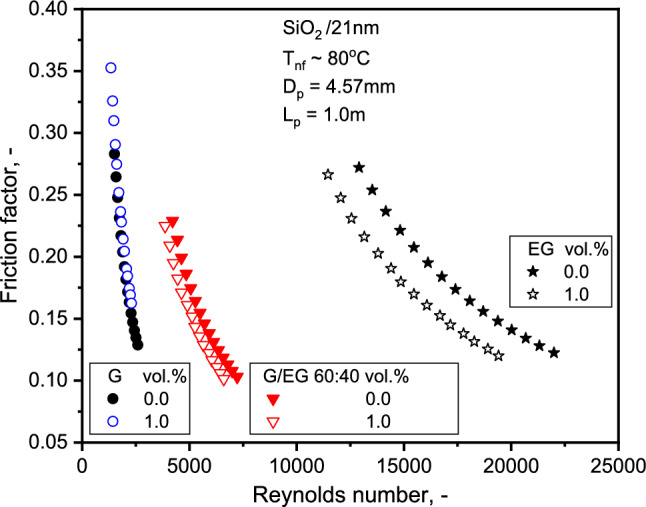
Comparison of FF for the three NFs with Re.

Figure 7 depicts a comparison of base liquid Nu with single-phase theory. As observed, the correlation closely predicts the Nu of the base liquids. The average absolute values of the deviations between Del Giudice et al.^42^ and Muzychka and Yovanovich^43^ from the experimental data are 1.6 and 3.8% for SiO_2_-EG, respectively, while the deviations are 0.9 and 6.8% for SiO_2_-G NFs. The variation of the observed values from those estimated with Muzychka and Yovanovich's^43^ correlation increased with the Re. At a Re of 22,000, for instance, a maximum absolute deviation of 1.84% was determined. As the results show, the correlations for single-phase flow can be used to forecast the base liquid HTC with the slightest deviation. Similar experimental evidence can be found in the work of Hwang et al.^46^. The FF variation with the Re is illustrated in Fig. 8. The FF decreases marginally with concentration and significantly with the Re. The FF of 20.6% decrement with SiO_2_-EG and 4.6% increase with SiO_2_-G NFs with 1.0% NF compared to base liquid at 13,000 and 2000 Re, respectively. The decrease in SiO_2_-EG nanofluid FF might be due to the turbulent nature of flow as compared to SiO_2_-G at 1.0% concentration. At 1.0% concentration, the viscosity of SiO_2_-G is approximately 10 times greater than SiO_2_-EG, and flowing in the laminar range of Re might be the reason for enhancement in FF.

Further, the glycerol experiences greater friction within its adjacent fluid layers and exerts excellent flow resistance than ethylene glycol on external energy exposure. Also, the glycerol molecules' chemical (hydrogen) bonding is significantly robust, which means more external energy would be needed to break the intermolecular attraction forces and cause the liquid particles to move. A theoretical analysis was undertaken following the technique explained by Sharma et al.^30^ to understand the flow characteristics in detail.

**Figure 9:**
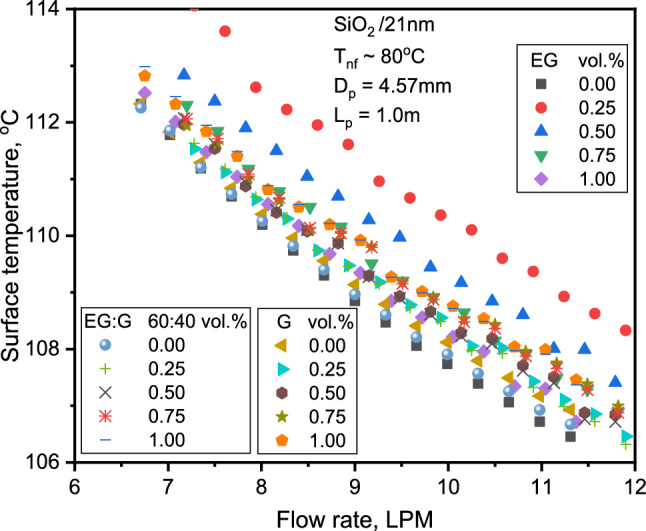
Variation of tube surface temperature with flow rate for the three NFs.

**Figure 10:**
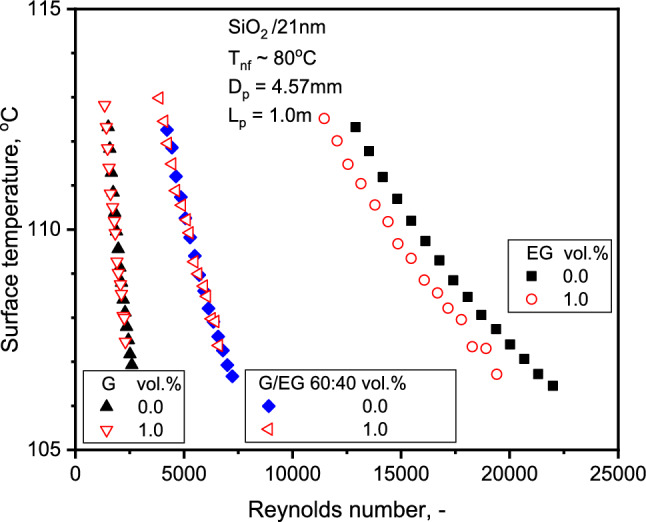
Comparison of tube surface temperature for base liquid and NFs at 1.0% concentration.

From the Figs. 9 and 10, the surface temperature decreases with increasing flow rate and Re. Figure 9 shows the surface temperature variation with flow rate for SiO_2_-G and SiO_2_-EG NFs. The wall temperature of SiO_2_-G is comparatively lower than SiO_2_-EG NFs for specific concentrations. The NF heat capacity of SiO_2_-G is approximately 17% greater than SiO_2_-EG, which might be the cause for the lower wall temperatures observed. Also, the flow velocities with G are lower than with EG, which might be another reason for lower wall temperatures with SiO_2_-G. The surface temperature does not vary significantly for SiO_2_-G compared to SiO_2_-EG NF with Re in Fig. 10.

**Figure 11:**
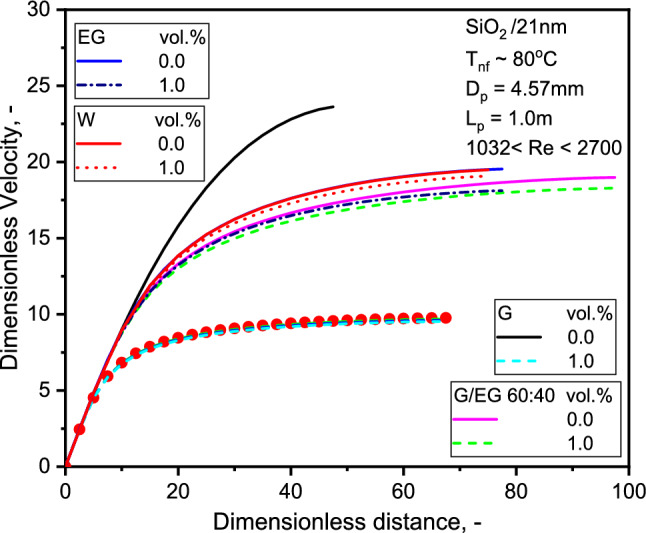
Variation of dimensionless velocity with radial distance for the three NFs.

**Figure 12:**
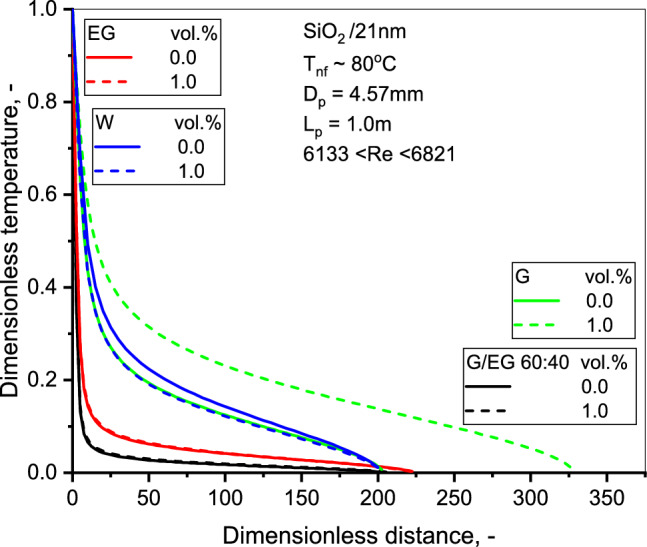
Variation of dimensionless temperature with radial distance for the three NFs.

The effect of concentration has been investigated for the evolution of nondimensional flow velocity with dimensionless length, as shown in Fig. 11, established in the earlier work by Sharma et al.^51^. The velocity profile of SiO_2_-G is relatively flatter than SiO_2_-EG NFs. Albeit, the flattening of dimensionless velocity was more pronounced with SiO_2_-G NF owing to the motion of the NPs, as compared to the base liquid. NPs can move either towards the tube wall or the axis region, depending on the magnitude of the density ratio of the NPs to the base liquid. The velocity profile flattens as the NPs move more rapidly than the fluid; the NPs migrate toward the tube wall. When the fluid moves quickly, the particles drift toward the axis of the tube^46,51^. Figure 12 display the predicted dimensionless temperature distribution as a function of dimensionless distance. As can be seen, the temperature decreases with concentration.

**Figure 13:**
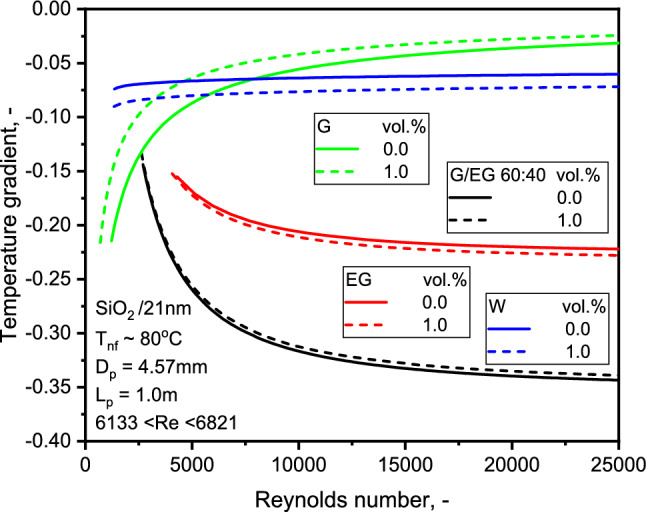
Comparison of temperature gradient with flow Re for the three NFs.

The NF temperature profile of SiO_2_-G NF shows a higher temperature than the base liquid profile. The NF is associated with a decreasing temperature gradient in the flow vicinity away from the tube surface. One may compare the temperature gradients of SiO_2_-EG and SiO_2_-G nanofluids as illustrated in Fig. 13. Results show a logarithmic growth of the temperature gradient as the Re increase for SiO_2_-G NFs. At the same time, the SiO_2_-EG NFs display an inverse trend. More striking was the increasing temperature gradients for SiO_2_-G NF. The Nu does not vary significantly for the G and EG-based NFs. These findings concord with the earlier observation of higher HTCs with low-viscosity NFs^10^.

### Machine learning-based model prediction

The experimental data and results collected in the experimental analysis part were used to create predictive models for friction factor and Nu number. The dataset typically contains information about Reynold’s numbers and Prandtl numbers estimated from the test setting and results. This data serves as the foundation for training and testing the predictive models. Often, it is necessary to preprocess raw experimental data to eliminate missing numbers, outliers, and other issues^52,53^. For preparing the dataset for model training, Python tools such as Pandas are utilized to clean and organize it. Understanding the structure of the dataset is crucial in order to gain valuable insights. With Python libraries such as Matplotlib and Seaborn, visualizations become effortless. This allows one to explore the connection between various factors, identify trends, and gain insights into the underlying patterns within the data.

#### Data pre-analysis

**Figure 14:**
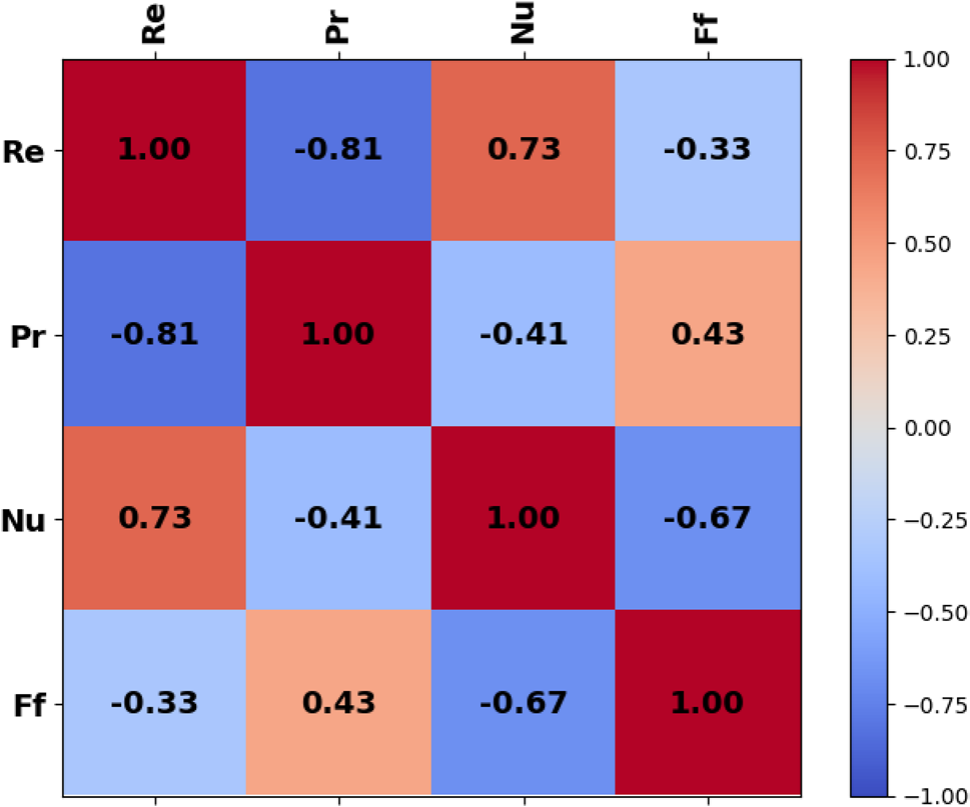
Correlational heatmap.

The correlation heatmaps for the data used in this study are shown in Fig. 14. It can be observed that There is a clear positive correlation between the Reynolds number and the Nusselt number, having a correlation coefficient of 0.73. This indicated that increasing the Re number will help in increasing the rate of heat transfer. On the other hand, a correlation coefficient of − 0.33 was observed between Re numbers and friction factors, indicating a negative correlation. The Prandtl number indicates a negative correlation (− 0.41) with the Nu number and a positive correlation (0.43) with the FF.

The descriptive statistical as listed in Table 3, offer insights into the characteristics of the dataset. For the variable Re number, it was noted that a mean value of 8154.069 and a standard deviation of 6569.183. The IQR spans from the 25th percentile at 2209.716 to the 75th percentile at 14,498.13. In the case of Re a negative kurtosis was observed, indicating a slightly flatter shape compared to a normal distribution. In the case of Pr number, a mean value of 117.947 and a standard deviation of 94.784 were observed. The IQR spans from 26.614 to 246.968, with a median value (50th percentile) of 80.166. The kurtosis for Pr is negative, indicating a distribution that is slightly flatter than the normal distribution^54,55^.

**Table 3:** Descriptive statistical values.

	Re	Pr	Nu	Ff
Count	225	225	225	225
Mean	8154.069	117.947	28.115	0.176
Std	6569.183	94.784	2.288	0.052
Minimum	1342.382	24.586	23.336	0.095
25%	2209.716	26.614	26.431	0.135
50%	5654.911	80.166	28.013	0.168
75%	14,498.13	246.968	29.549	0.208
Maximum	21,991.98	255.744	33.604	0.353
Kurtosis	− 1.042	− 1.498	− 0.518	0.193
Skewness	0.705	0.545	0.237	0.766

On the other hand, in the case of the Nu number, the mean was estimated at 28.115, and the standard deviation of 2.288. The kurtosis value in the case of Nu was also negative, suggesting a distribution that is slightly flatter than normal. Also, in the case of friction factor, a mean value of 0.176 was observed a standard deviation of 0.052. The IQR spans from 0.135 to 0.208, with a median value of 0.168. However, in the case of Ff is positive, demonstrating a slightly peaked distribution compared to the normal distribution.

Overall, these descriptive statistics provide a comprehensive overview of the dataset, including measures of central tendency, dispersion, and shape of the distributions for each variable.

#### Nusselt number model

**Figure 15:**
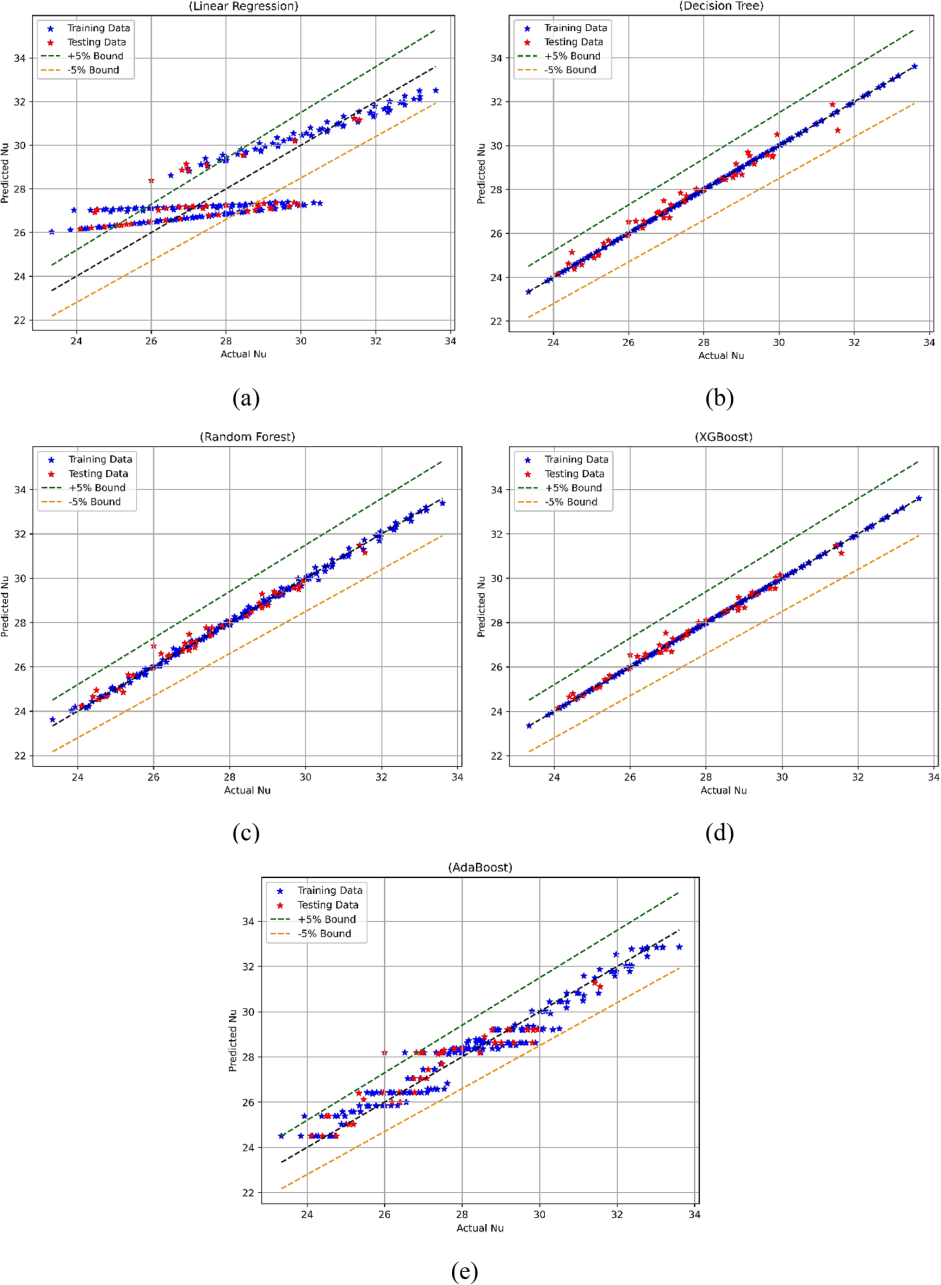
Nu model’s actual vs predicted performance for (**a**) LR, (**b**) DT, (**c**) RF, (**d**) XGBoost, (**e**) AdaBoost.

A predictive model for the Nu number was built following the completion of the data analysis, which included the use of a correlation heatmap and descriptive statistical analysis. A random split of the data was performed at a ratio of 70:30 for the purpose of training and testing the model. The five ML approaches LR, RF, XGBoost, AdaBoost, and DT were employed for the development of prediction models. Following the completion of the models, they were utilized for the purpose of prediction. At the end of the Nu number models, the comparative findings depicting actual vs predicted Nu results are displayed in Fig. 15a–e. Figure 15a demonstrates the contrast between the actual values and the values predicted for LR-based model, and Fig. 15b for DT, Fig. 15c for RF, Fig. 15d for XGBoost, and the Fig. 15e for AdaBoost. It can be observed that except LR all other models performed in a satisfactory manner; however, the XGBoost-based model was more superior than the other models^56–58^.

The statistical evaluation of the Nu models developed with various methods was conducted and the results are listed in Table 4. In case of LR-based model a training MSE of 1.9 and a test phase MSE of 2.26 were observed. This indicates a poor level of performance indicating that the error caused by LR is substantially larger in comparison to that of other models. Given that the R^2^ values for LR are 0.651 for training and 0.35 for testing, it may be inferred that LR only explains a moderate amount of the variance in Nu. In the case of DT-based model, it showed a flawless performance, demonstrating MSE as 0 and R^2^ as 1. These values indicates that there were almost nil prediction errors. The DT-based Nu model demonstrated stellar performance during model testing also. It displayed a Test MSE of 0.095 and a Test R^2^ of 0.972^59,60^.

**Table 4:** Statistical evaluation results of the Nu model.

Model	Train MSE	Test MSE	Train R^2^	Test R^2^	Train Wi	Test Wi	Train MAPE	Test MAPE
LR	1.9	2.26	0.651	0.35	0.888	0.726	4.156	4.71
DT	0	0.095	1	0.972	1	0.993	0	0.908
RF	0.0108	0.069	0.998	0.98	0.999	0.995	0.283	0.714
XGBoost	0.00001	0.045	0.9999	0.9871	0.9999	0.9967	0.0094	0.5851
AdaBoost	0.276	0.4451	0.9496	0.8725	0.9862	0.964	1.52	1.862

The performance of RF-based models was good as it had a train MSE of 0.0108 and a Test MSE of 0.069, indicating only a few errors throughout both the training and testing phases of the model development process. The R^2^ values in the case of RF were fairly high, at 0.998 during training and 0.98 for testing. This shows that RF may capture a significant portion of the volatility in Nu. Furthermore, XGBoost performs very well, as indicated by its Train MSE of 0.00001 and Test MSE of 0.045, both of which imply a low number of errors. With a training value of 0.9999 and a testing value of 0.9871, the XGBoost-based model’s R^2^ values are very high, suggesting that the model has an excellent link to the data. Given that AdaBoost has a Train MSE of 0.276 and a Test MSE of 0.4451, indicating that it has few mistakes, its performance is fairly excellent. AdaBoost's R^2^ values of 0.9496 for training and 0.8725 for testing indicate that the model fits the training data well, while the test data shows a minor reduction in performance^61–63^.

It can be observed that both RF and XGBoost stand out as the best models for predicting the Nu model, on the basis of statistical evaluations. This is owing to the fact that they produce low error metrics and high R^2^ values for both the training and test datasets. Because these models can correctly capture the complexities of the data and generate accurate predictions for the Nu model, they are suitable for regression applications.

**Figure 16:**
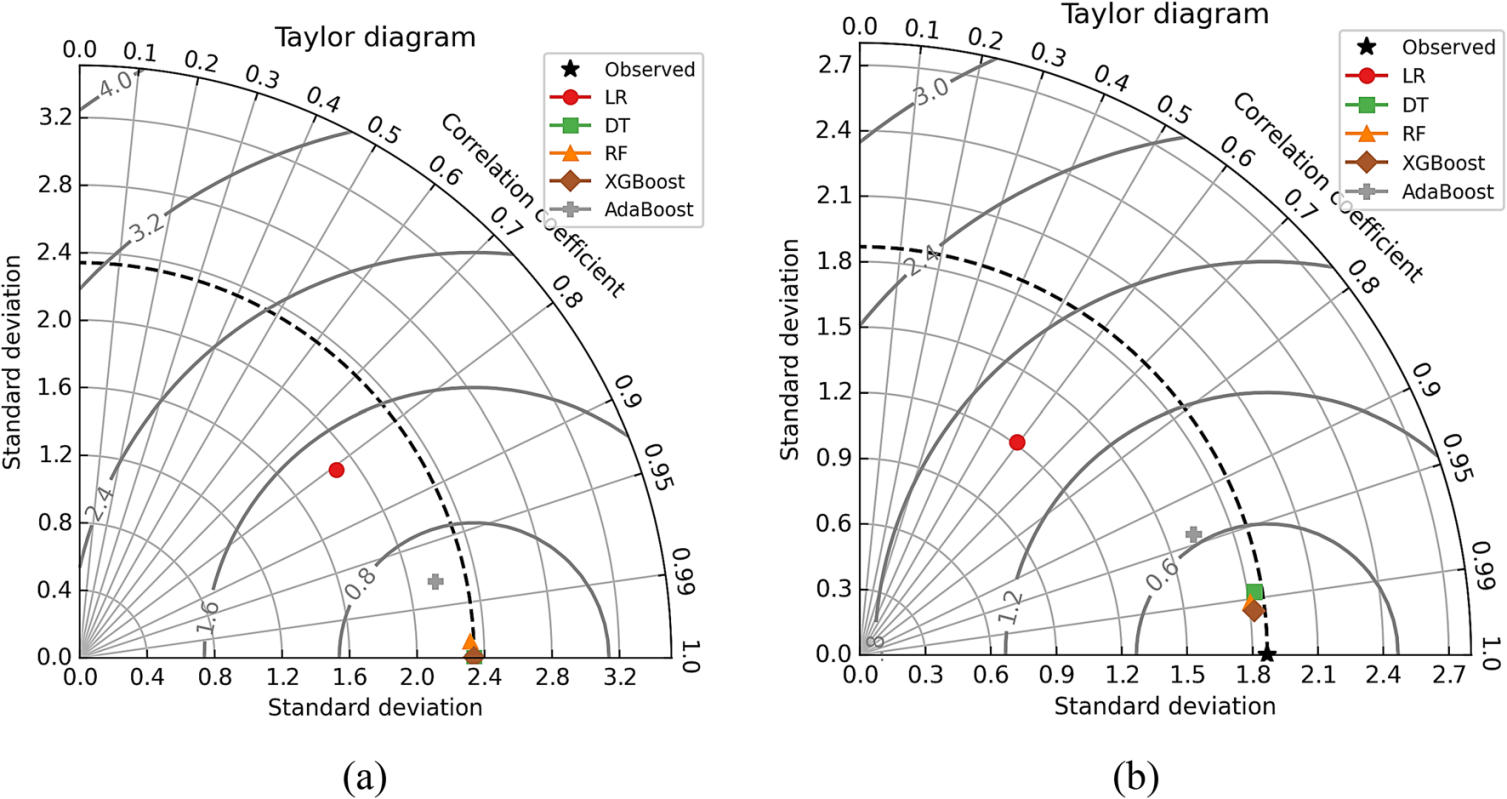
Taylor’s diagram for Nu model during (**a**) training and (**b**) testing phase.

**Figure 17:**
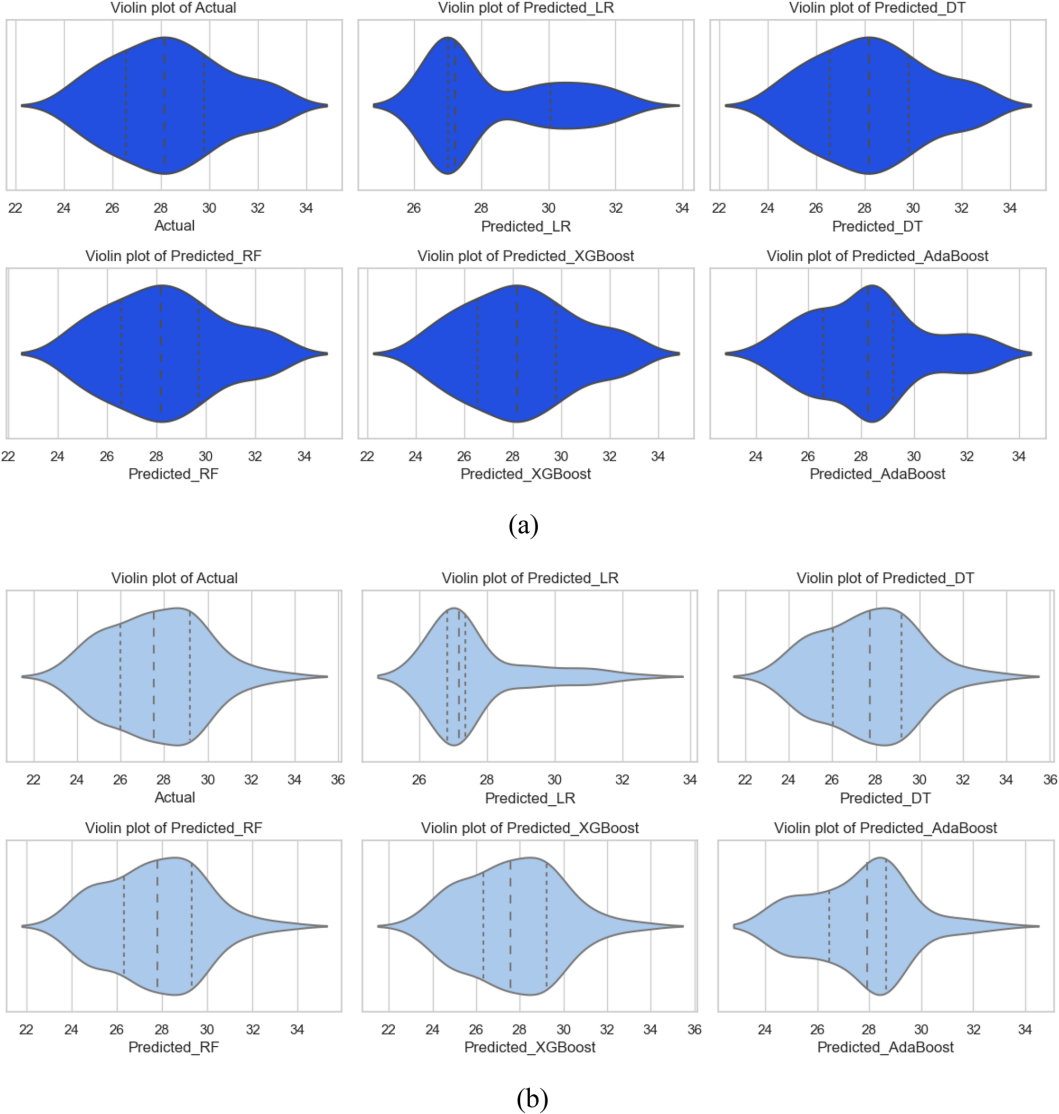
Violin plots for Nu model for (**a**) training and (**b**) testing phase.

The models were further tested using visual description by employing Taylor’s diagram and violin plots to compare their performance. Figure 16 depicts Taylor’s diagram while the violin plots for all models are depicted in Fig. 17. In the case of Nu model prediction during training, it can be observed that both DT and XGBoost-model performed superior to other models but the XGBoost-based model was best. Similarly, in the case of model testing, the XGBoost was the best model among the five-model tested in this case. The improved performance of RF and XGBoost models is primarily attributable to their robustness in dealing with complex, nonlinear interactions, as well as their ability to prevent overfitting using ensemble approaches. The violin plots were drawn for each of the models as depicted in Fig. 17a for the training phase while Fig. 17b shows violin plots for the testing phase. Hereto, it could be observed that the XGboost–based model was superior to other models as can be observed by the shape of violin plots as well as median lines on the plots.

#### Friction factor model

**Figure 18:**
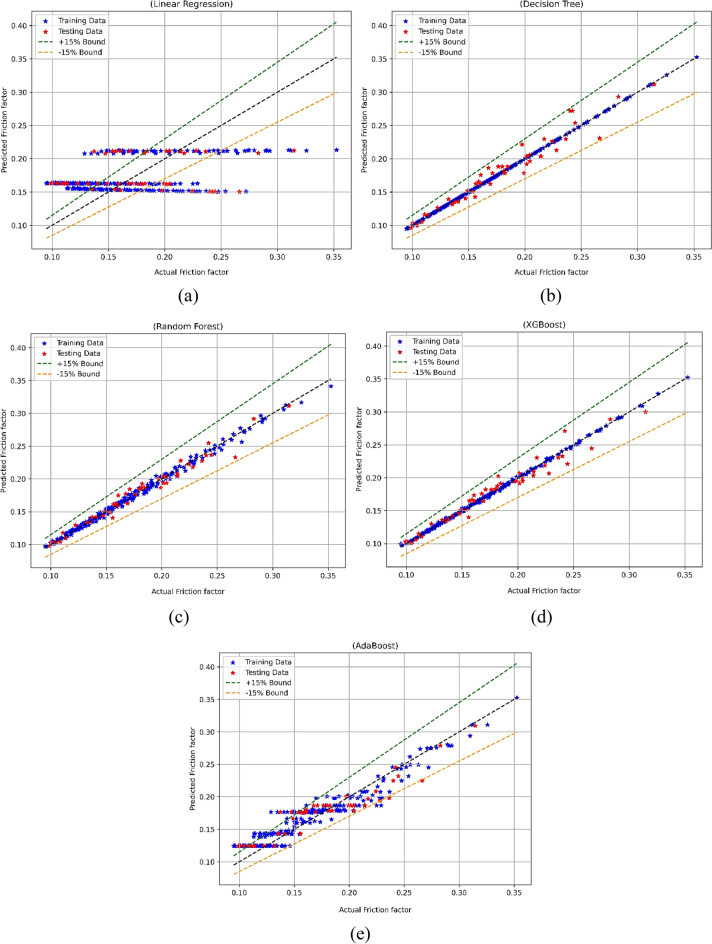
Friction factor model’s actual vs predicted performance for (**a**) LR, (**b**) DT, (**c**) RF, (**d**) XGBoost, (**e**) AdaBoost.

In the case of friction factor also, the five machine learning algorithms (LR, RF, XGBoost, AdaBoost, and DT) were employed for the creation of prediction models. After the models were completed, they were used to make predictions. Figure 18a–e show the comparison findings from the Nu number models, illustrating real vs expected Nu outcomes. Figure 18a shows the difference between the actual and predicted values for the LR-based model, followed by Fig. 18b for DT, 18c for RF, 18d for XGBoost, and 18e for AdaBoost. Except for LR, the other models performed satisfactorily; nonetheless, the XGBoost-based model outperformed the others.

The statistical evaluation of the friction factor models built using various approaches was performed, and the results are presented in Table 5. In the case of an LR-based model, the training MSE was 0.0022, while the test phase MSE was 0.00252. This shows a low degree of performance, implying that the error generated by LR is significantly bigger than that of other models. Given that the R^2^ values for LR are 0.232 for training and − 0.022 for testing, it can be concluded that LR only explains a small fraction of the variance in friction factor. The DT-based model performed flawlessly, with an MSE of zero and an R^2^ of one. These figures suggest that there were virtually no forecast mistakes. The DT-based Nu model performed admirably throughout model testing as well. It had a Test MSE of 0.00014 and an R^2^ of 0.94.

**Table 5:** Statistical evaluation results of the FF model.

Model	Train MSE	Test MSE	Train R^2^	Test R^2^	Train Wi	Test Wi	Train MAPE	Test MAPE
LR	0.0022	0.00252	0.232	− 0.022	0.612	0.51	23.27	23.999
DT	0	0.00014	1	0.94	1	0.985	0	4.758
RF	0.00001	7.00E−05	0.994	0.97	0.998	0.992	1.84	3.45
XGBoost	0.000002	0.0001	0.999	0.958	0.9998	0.9888	0.67	4.142
AdaBoost	0.00026	0.00036	0.906	0.852	0.9725	0.952	8.436	9.747

RF-based models performed well, with a train MSE of 0.00001 and a test MSE of 0.00007, indicating only a few errors during the model generation process. The R^2^ values for RF were rather high, at 0.994 during training and 0.97 during testing. This suggests that RF may capture a considerable percentage of the volatility in Nu. Furthermore, XGBoost works admirably, as seen by its Train MSE of 0.000002 and Test MSE of 0.0001, both of which suggest a smaller error. The R^2^ values for the XGBoost-based model are extremely high, with a training value of 0.999 and a testing value of 0.958, indicating that the model has a strong relationship to the data. Given that AdaBoost has a Train MSE of 0.00026 and a Test MSE of 0.00036, showing that it makes few errors, its performance is rather good. AdaBoost's R^2^ values of 0.906 for training and 0.852 for testing suggest that the model fits the training data well, while the test data reveals a modest loss in performance.

Statistical studies show that both RF and XGBoost are the best models for predicting the Nu model. This is due to the fact that they provide low error metrics and strong R^2^ values across both the training and test datasets. These models are appropriate for regression applications because they can accurately capture data complexity and give reliable predictions for the Nu model.

**Figure 19:**
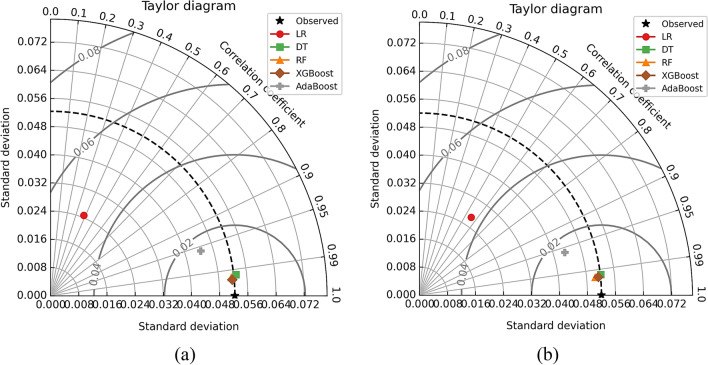
Taylor’s diagram for friction factor model during (**a**) training and (**b**) testing phase.

**Figure 20:**
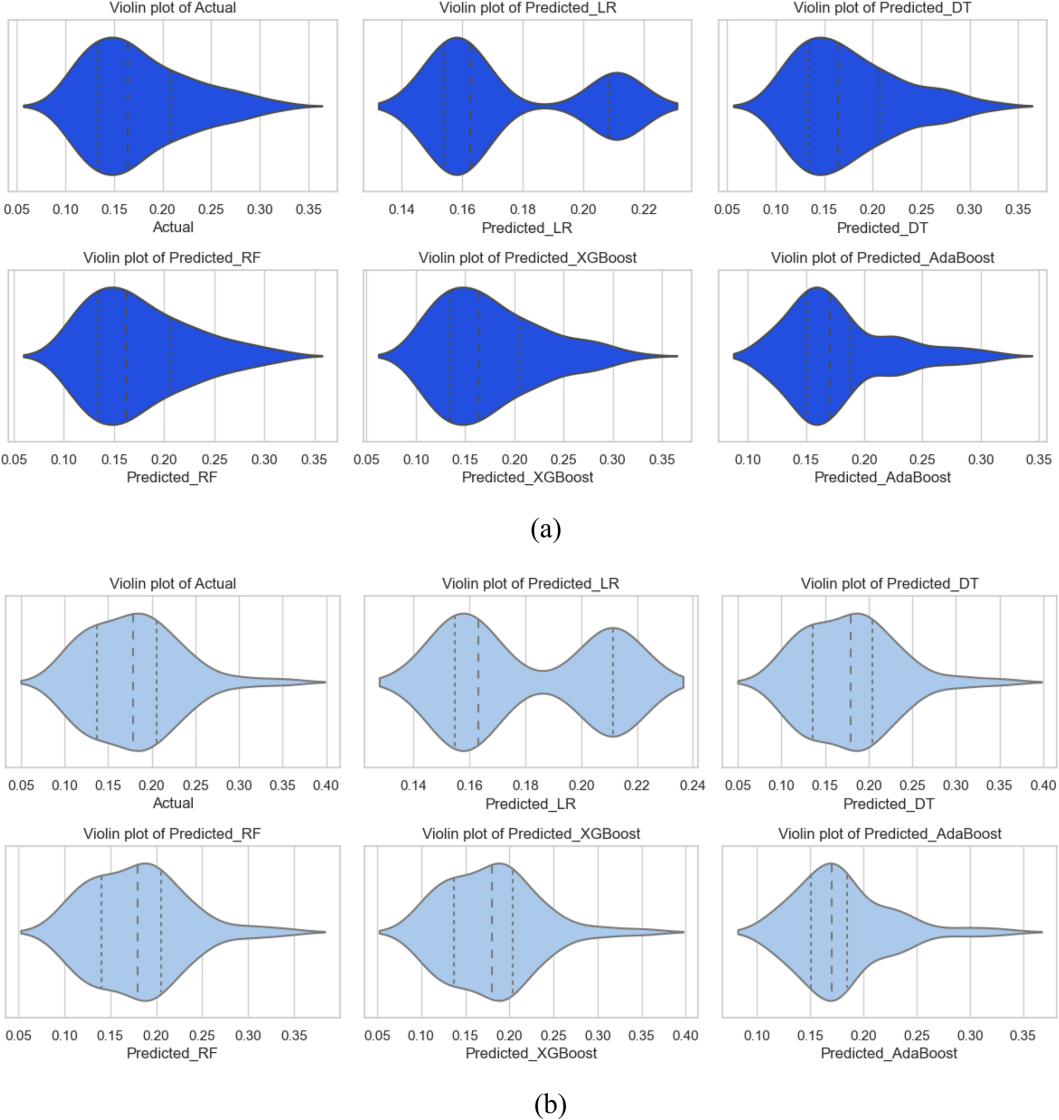
Violin plots for friction factor model for (**a**) training and (**b**) testing phase.

The models were further examined visually using Taylor's diagram and violin plots to compare their performance. Figure 19 illustrates Taylor's diagram, and Fig. 20 depicts the violin plots for all models. In terms of friction factor model prediction during training, both the RF and the XGBoost models outperformed the other models, but the XGBoost-based model was the best. Similarly, in terms of model testing, the XGBoost outperformed the other five models evaluated. Violin plots were constructed for each model as shown in Fig. 20a for the training phase, and Fig. 20b for the testing phase. Previously, it was noted that the XGBoost-based model outperformed other models, as seen by the shape of the violin plots and the median lines on the plots.

## Conclusions

The work presents a convective heat transfer coefficient and friction factor assessment of ethylene glycol and glycerol-based non-porous silicon dioxide nanofluid flow in a tube under constant heat flux boundary conditions. The experiments were conducted for Reynolds numbers between 1300 and 21,000 and concentrations ranging from 0 to 1.0% volume at approximately 80 °C. The heat transfer coefficient and friction factor data were analyzed based on experimental thermophysical properties correlations. To model and predict the complex and nonlinear data acquired during experiments, prognostic model development using five machine learning techniques was employed. The following are the main results of the study:

- Maximum heat transfer enhancements of 5.9% and 1.9% were determined at a 12 L per minute flow rate of silicon dioxide-ethylene glycol in the turbulent and silicon dioxide-glycerol nanofluids in the laminar range of Reynolds numbers for 1.0% concentration at approximately 80 °C.
- Factors such as thermal conductivity enhancement, particle migration, Brownian motion of nanoparticles, and thinning of boundary layer thickness possibly play an important role in heat transfer augmentation. Single-phase flow correlations can predict the Nusselt number of silicon dioxide-ethylene glycol and silicon dioxide-glycerol nanofluids favorably.
- The friction factor decreases with concentration for silicon dioxide-ethylene glycol nanofluid and the Reynolds number. Turbulent flow silicon dioxide-ethylene glycol yields lower friction factor values than laminar flowing silicon dioxide-glycerol nanofluids with 1.0% volume. Silicon dioxide-glycerol nanofluids presented lower surface temperatures, lower temperature gradients, and flattened velocity profiles.
- Random forest-based models had good performance, with a training mean squared error of 0.0108 and a test mean squared error of 0.069. Extreme gradient boosting performed well, with a training mean squared error of 0.00001 and a test mean squared error of 0.045, implying a low number of errors.
- Both random forest and extreme gradient boosting were the best models for predicting the Nusselt number model, owing to their low error metrics and high R-squared values. Models were further tested using visual descriptions, with extreme gradient boosting being the best model in both training and testing.
- Random forest-based models performed well, with a training mean squared error of 0.00001 and a test mean squared error of 0.00007, indicating few errors during the model generation process. Extreme gradient boosting performed admirably, with a training mean squared error of 0.000002 and a test mean squared error of 0.0001, suggesting a smaller error.
- Adaptive boosting's performance was good, with a training mean squared error of 0.00026 and a test mean squared error of 0.00036, indicating a good fit to the training data. Random forest and extreme gradient boosting were the best models for predicting the Nusselt number model due to their low error metrics and strong R-squared values across both the training and test datasets.
- Visual examination of the models using Taylor's diagram and violin plots showed that the extreme gradient boosting-based model was the best in terms of friction factor model prediction during training and in terms of model testing.

## Data Availability

The data is available within the manuscript.
